# The Effects of Surface Patterning and Photobiomodulation on the Osseointegration of Titanium Implants in Osteoporotic Long Bones: An In Vivo Study in Rats

**DOI:** 10.3390/jfb15110346

**Published:** 2024-11-14

**Authors:** Theodor Popa, Mircea Negrutiu, Luciana Madalina Gherman, Alina Deniza Ciubean, Dan Ionut Cosma, Dan Gheban, Catalin Popa, Laszlo Irsay

**Affiliations:** 1 Rehabilitation Hospital, Str. Viilor 46-50, 400347 Cluj-Napoca, Romania; spitalulclinicderecuperare@gmail.com (T.P.); alina.ciubean@umfcluj.ro (A.D.C.); dcosma@umfcluj.ro (D.I.C.); laszlo.irsay@umfcluj.ro (L.I.); 2 Department of Rehabilitation, “Iuliu Hatieganu” University of Medicine and Pharmacy, Str. Victor Babes nr. 8, 400012 Cluj-Napoca, Romania; 3 Department of Orthopedics, “Iuliu Hatieganu” University of Medicine and Pharmacy, Str. Victor Babes nr. 8, 400012 Cluj-Napoca, Romania; 4 Department of Dermatovenerology, “Iuliu Hatieganu” University of Medicine and Pharmacy, Str. Victor Babes nr. 8, 400012 Cluj-Napoca, Romania; negrutiu.mircea.ionut@elearn.umfcluj.ro; 5 Dermatovenerology, Emergency County Hospital, Str. Clinicilor nr. 3-5, 400006 Cluj-Napoca, Romania; 6 Experimental Centre, “Iuliu Hatieganu” University of Medicine and Pharmacy, Str. Victor Babes nr. 8, 400012 Cluj-Napoca, Romania; luciana.gherman@umfcluj.ro; 7 Department of Anatomical Pathology, “Iuliu Hatieganu” University of Medicine and Pharmacy, Str. Victor Babes nr. 8, 400012 Cluj-Napoca, Romania; dan.gheban@elearn.umfcluj.ro; 8 Department of Materials Science and Engineering, Technical University of Cluj-Napoca, 103-105, Muncii Ave., 400641 Cluj-Napoca, Romania

**Keywords:** surface-patterned titanium implants, photobiomodulation, osteoporosis, LASER therapy

## Abstract

This study aimed to assess the impact of titanium surface patterning used in combination with photobiomodulation therapy on enhancing osseointegration in osteoporotic bone fractures. C.p. titanium implants were employed, half with an unmodified surface and half with a modified one, showing a nanostructured cellular surface. Surface patterning aimed to obtain a complex morphology designed for better osseointegration, using a selective anodization process after photoresist coating. A total of 52 rats were used, of which 4 were sacrificed 12 weeks after ovariectomy to evaluate bone density. A total of 48 rats received titanium implants in both tibiae and underwent surgery for implant placement and bone fracture. Half of the rats were subjected to photobiomodulation. The times of sacrifice were 2, 4, and 6 weeks after finalizing LASER therapy. The evaluation methods were micro-CT scanning, the mechanical pull-force test, and morphology. Mechanical tests revealed a significant difference in the surface-patterned titanium with the LASER group at 2 weeks, but not at 4 and 6 weeks. This group outperformed regular titanium and titanium/LASER groups. Micro-CT showed no significant differences, while the morphology indicated better bone quality at 4 weeks in all LASER-treated groups. The effect of surface patterning and photobiomodulation leads to better osseointegration, especially in the earlier stages.

## 1. Introduction

Bone fractures can occur in all patients, but the probability is higher if there are underlying conditions that reduce bone density. Osteoporosis is a systemic skeletal disease characterized by reduced bone density, increased fragility, and a higher risk of fracture [1]. Menopause and old age are some of the most common causes of osteoporosis. The most common sites of fragility fractures are the vertebrae, proximal femur, distal radius, proximal humerus, and pelvis [2]. Fractures could lead to loss of function, disability, acute pain, and the exacerbation of underlying comorbidities. Advancements in orthopedics and rehabilitation could improve quality of life and life expectancy by improving the bone/implant interaction, accelerating the healing time, and reducing the risk of complications. Improving bone mass faster in a certain area could lead to fewer complications and a faster recovery time. Low-level LASER therapy (LLLT), or photobiomodulation, is a physiotherapeutic treatment method employed by rehabilitation physicians to stimulate the angiogenesis, fracture healing, and osteogenic differentiation of stem cells [3]. The LASERS used in biostimulation emit within the red or infrared spectrum with a power range from 50 mW to 1 W [4].

Studies suggest that LASER therapy stimulates cytochrome c oxidase (CCO) through the photolysis of the inhibitory nitric oxide, boosting mitochondrial respiration and ATP production [3]. LLLT alters mitochondrial membrane potential, enhancing the cell’s redox state, increasing Fe²⁺ oxidation, inhibiting proline hydroxylases (PHDs), and deregulating HIF-1α. The rise in reactive oxygen species affects signaling pathways involved in cell proliferation, survival, repair, and regeneration [5]. In vitro studies conducted in the earlier phases of bone formation (inflammatory, angio-mesenchymal, and bone formation) show that the WNT/β-catenin pathway is key for bone healing by helping pre-osteoblasts develop into bone-forming cells. However, inflammation can block this process due to cytokines like TNF, a protein that inhibits WNT signaling. LASER therapy can reverse this by boosting β-catenin activity, speeding up bone repair [6]. The NF-kB pathway triggers inflammation, but LLLT has been shown to reduce its activity in stem cells, helping to control inflammation and supporting healing [7]. In the angio-mesenchymal phase, VEGF promotes angiogenesis, and LASER treatment enhances its expression, boosting blood vessel formation [5]. FGF aids osteoblast differentiation and cell growth, with photobiomodulation shown to increase its levels. PDGF supports mesenchymal stem cell (MSC) proliferation and angiogenesis, and LASER therapy significantly increases its expression [8]. Runx-2 is crucial for recruiting MSCs, promoting their differentiation into osteoblasts, and activating bone matrix genes like col1a1 and Osteocalcin [5]. LASER therapy has been shown to enhance MSC differentiation towards osteogenesis. GaAlAs diode LASER irradiation also increases bone marrow stromal cell (BMSC) proliferation and osteoblast differentiation. BMP/TGF-β pathways are key for osteogenesis, and photobiomodulation boosts BMP and TGF-β expression, promoting osteogenic differentiation via ROS activation [9].

In vivo, studies show that LASER therapy accelerates bone regeneration by reducing inflammation and enhancing angiogenesis, with lasers at 830 nm increasing VEGF expression and at 1064 nm boosting PDGF and FGF levels [10,11]. Photobiomodulation also promotes osseointegration and bone formation, increasing markers like BMP2 and OCN. Clinical trials confirm faster bone regeneration and fewer complications with LASER therapy, particularly at 830 nm and 660 nm wavelengths [5]. Titanium alloys are used in bone surgery because of their biocompatibility, which occurs due to low electrical conductivity and the formation of the thin passive oxide layer that is retained at pH values of the human body and does not permit oxidation [12].

At present, the prevention of post-implantation infections or excessive inflammation is achieved through drug administration, whether orally or intravenously. Although this strategy is usually effective, issues regarding low solubility, selectivity, bioavailability, or unwanted side effects may occur [13]. Also, the risk of titanium implant failure can be augmented by inappropriate biomechanics at the interface with bone tissue, leading to improper osseointegration [14]. This is why a surface treatment, in view of assuring an optimal chemistry/surface morphology, is required to increase titanium implants’ biocompatibility. Several methods are available at this moment: physical vapor deposition (PVD), chemical vapor deposition (CVD), sol–gel coating, electrochemical anodization, etc. Among these, electrochemical anodization is the easiest to conduct, as it employs a simple setup, is a low-energy consumer, and assures stable and compact surface titania, often in the shape of nanotubes [15]. Anodic oxidation of titanium can be performed in many types of electrolytes, such as sulfuric acid, hydrofluoric acid, acetic acid, chromic acid, or combinations of these, leading to different aspects of the surface [16]. Among these, HF is employed easily and yields titanium fluorides formed on top of the fresh titania layer. The electrochemical mechanism assures repetitive cellular morphology of titania, with hemispheric-shaped pores, which are found on the surface of the titanium. It is found that a controlled nanopattern consisting of hemispherical 51 ± 9 nm protrusions promotes early osteogenic differentiation and osteoblastic activity [17].

This study aimed to explore the impact of surface patterning on titanium implants combined with photobiomodulation for enhancing the osseointegration of commercially pure (c.p.) grade 1 titanium implants in long fractured osteoporotic bones. Implant performance was assessed using three primary methods: imaging, mechanical testing, and morphological analysis.

## 2. Materials and Methods

The study was conducted at the Experimental Facility of the University of Medicine and Pharmacy “Iuliu Hațieganu” Cluj-Napoca. The University’s Ethics Committee approved the protocol on 16.11.2022 (AVZ291), and the Sanitary Veterinary and Food Safety Agency authorized the project on 19.12.2022 (nr. 344). The animal experiments were performed on white female Wistar Rattus Norvegicus rats aged 4 months. Their health state was assessed by the veterinary doctor who supervised the experiments. After surgery, the animals were kept at 22 °C in a light–darkness cycle of 12 h and supplied with water and food ad libitum.

**Figure 1:**
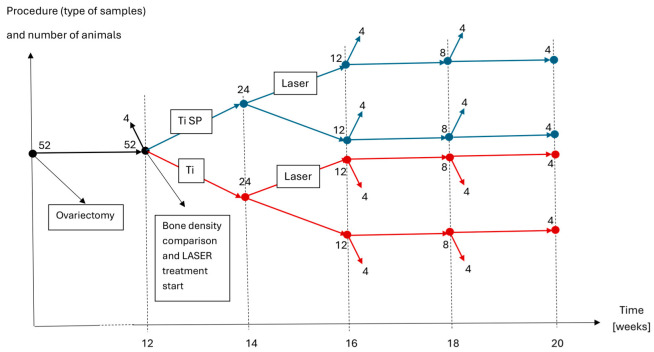
A flow chart of this study that shows the distribution of animals and intervention at a certain time (weeks 16, 18, and 20 correspond to the time of sacrifice at 2, 4, and 6 weeks after finalizing the LASER therapy protocol).

The number of rats used in the experiment was 52 as seen in Figure 1. In total, 50 of the animals underwent surgery for bilateral ovariectomy, followed by 3 months of rest. Moreover, 2 of the rats were not subjected to bilateral ovariectomy and were used as a control to evaluate bone density compared to the ovariectomized animals. After 12 weeks, 4 animals were sacrificed to assess bone density using micro-CT scanning (2 of the rats were ovariectomized), and the others were subjected to surgery. Each experimental animal received a titanium implant in both tibiae, followed by a fracture performed by the same physician. The implants were 1 mm in diameter and 10 mm long and made from c.p. titanium grade 1 provided by the Technical University of Cluj-Napoca’s Department of Materials Science and Engineering. Two types of implants were used during this research: half had a smooth surface, and the others were subjected to “surface patterning”. Each rat received the same type of implant and treatment in both tibiae. One rat died after the implantation surgery and was removed from this study. After surgery, half of all the rats were subjected to LASER therapy on both tibiae. The animals were categorized into 4 groups as follows: 1. titanium implants (Ti); 2. titanium implants with LASER therapy (Ti+L); 3. surface-patterned titanium implants (TiSp); 4. surface-patterned titanium implants with LASER therapy (TiSp+L). The animals that underwent photobiomodulation therapy were exposed to a low-level LASER for 14 days, once every 48 h, for 7 sessions. In total, 4 animals from each group were sacrificed 2, 4, or 6 weeks (2w, 4w, 6w) after finishing the LASER stimulation. One rat from the Tisp+L group died during anesthesia on the 5th day of photobiomodulation, 10 days after surgery, and was removed from the experiment. The methods of evaluation were micro-CT scan, morphology, and pull-force tests.

### 2.1. Implant Surface Patterning

**Figure 2:**
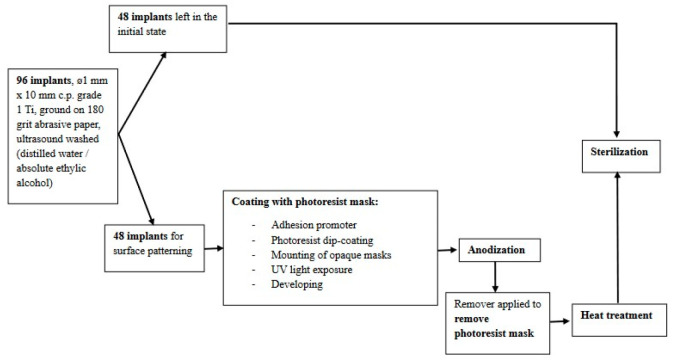
A flow chart of the implant preparation process.

**Figure 3:**
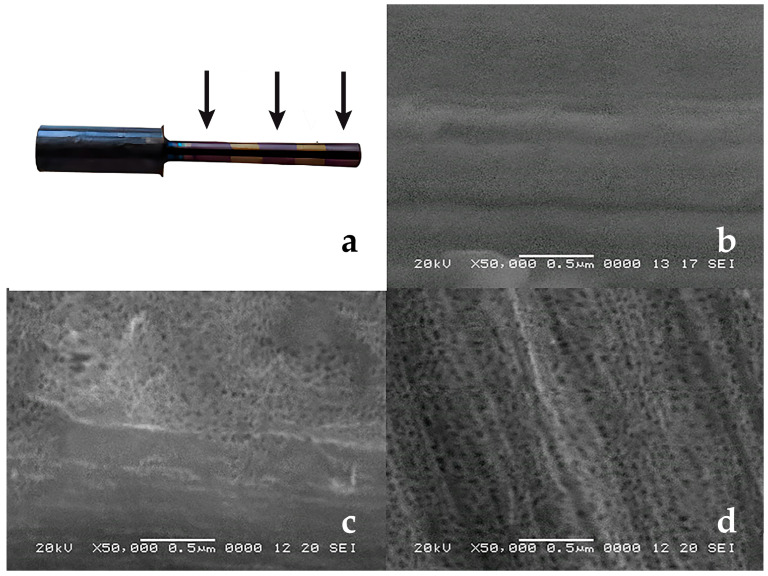
The implant surface was observed through scanning electron microscopy (SEM) (JEOL 5600 LV): (**a**) shows the implant with a technical means for gripping that was removed afterward, where the arrows point towards the “surface-modified” areas; (**b**) shows a smooth unetched surface; (**c**) shows the transition zone; and (**d**) shows the anodized area.

Surface patterning was performed to obtain an alternating morphology along the implant axis, alternating between anodically etched titania cellular zones and smooth ones (Figure 2). The idea was to offer the conditions for a strong fixation in bone regarding possible pulling-out stresses. Grade 1 c.p. Ti samples of ø1 mm × 10 mm were ground on 180 grit abrasive paper and washed in an ultrasound bath first in distilled water and then in absolute ethylic alcohol. Subsequently, the surfaces of half of the samples were coated with a photoresist layer (Positive Photoresist AR-P 325, Allresist GmbH, Strausberg, Germany), while the other half of the samples were used without any surface treatment. Previously, the samples to be patterned were conditioned with the adhesion promoter AR 300-80 new, Allresist GmbH, for 30 min in fumehood and treated at 70 °C for 30 min. The positive photoresist was dip-coated in two layers with a withdrawing speed of 10 mm/min, then heat-treated at 90 °C for 30 min. UV-opaque ring-shaped masks were mounted equidistantly on the samples, leaving equal masked/unmasked regions. Subsequently, the samples were exposed to UV light (Blue Wave 50, Dymax, Salt Lake City, UT, USA) for 90 s. The photoresist was removed from UV-exposed regions using Developer AR300-26, Allresist GmbH. Subsequently, the samples were subjected to anodization in a glass beaker, using 0.5% HF aqueous solution as the electrolyte and a stainless steel cathode, at 13 V d.c. for 3 min. After anodization, the photoresist rings that protected against anodic oxidation were removed with Remover AR300-76, Allresist GmbH. Subsequently, the samples were heat-treated in the oven starting from room temperature, with a 15 °C/min heating speed, and kept at 500 °C for 10 min, followed by slow cooling. The morphologies of the samples can be seen in Figure 3. The anodized area shows a nanostructured cellular morphology, with an inner diameter of around 50 nm, while the transition towards the smooth area is very well marked. Also, the transition zone shows that anodization produced a change in the overall level of the implant’s surface, an effect that was targeted for increasing the adhesion force at the implant–bone interface. Before being placed in the tibiae, all implants were immersed in 99% alcohol, dried, and placed in a UV chamber for sterilization.

### 2.2. Ovariectomy

The rats were anesthetized with a cocktail of Ketamine 10% and Xylazine 2% intramuscularly in the thigh using a hypodermic needle syringe. The abdomen of the rats was shaven and a median incision of 1.5 cm was made. The ovaries were manually found by following the fallopian tube from its emergence in the uterus. The ovaries were removed using an ophthalmic electric scalpel with a 15 × 180 mm tip. The abdominal muscles were separately sutured using absorbable stitches, followed by the suturing of the tegument.

### 2.3. Implant Placement and Fracture

The rats were anesthetized using a consistent mixture of Ketamine 10% and Xylazine 2%. The lower limbs were shaved with an electric shaver. A central incision was made along the patellar tegument, and an 18 G needle was used to create an entry point in the tibial plateau for implant insertion. The implant was securely press-fitted into the tibia, and the skin was sutured. A secondary incision was made along the outer side of the tibia, where the muscle tissue was gently separated to expose the bone without trauma. The same examiner then applied surgical pliers to create a controlled fracture in the metaphyseal region of the tibia, just below the fibular head, ensuring that the fibula remained intact.

### 2.4. LASER Protocol

LASER therapy was administered to the rats in groups Ti+L and TiSp+L on both tibiae. To minimize reflection from any remaining fur, the tibial area was first clipped with electric trimmers and then treated with shaving cream. The first LASER treatment was given 24 h post-surgery, with a total of seven sessions being conducted every 48 h over 14 days. Before each session, the rats were given small doses of Ketamine and Xylazine (0.05 mL/0.02 mL) to enhance comfort and prevent injury. A Diode LASER was used, delivering a continuous beam with 0.4 W power and a 830 nm wavelength. Each tibia received 80 J of energy from two points (40 J/cm^2^ per point), targeting both the internal and external sides of the fracture for 3 min and 20 s per tibia over a 1 cm^2^ area. This resulted in 80 J per tibia per session, totaling 560 J per tibia and 1120 J per rat across all sessions. No burns were observed during or after treatment.

### 2.5. Tissue Sampling and Methods of Assessment

The rats were euthanized 2, 4, and 6 weeks after the last session of LASER therapy. Both tibias of the rats were removed, cleaned, and preserved in formaldehyde 10%.

### 2.6. Micro-CT Scan

One tibia was selected at random from each rat and sent for a micro-CT evaluation. The micro-Ct analysis was performed using the SKYSCAN 1172 X-RAY Mi-cryotomography tool made by Bruker (Belgium). The scan slice thickness obtained was 13.6 μm. The scan results were interpreted using CTAn software (1.13.0.0, Bruker, Kontich, Belgium).

#### 2.6.1. Bone Density Evaluation

The bone density of the rats was observed through a micro-CT analysis by comparing 8 tibias from 4 rats. Two of the rats were non-ovariectomized. The rats were all females, with a similar weight and age, and were sacrificed 12 weeks after the ovariectomy. The tibial diaphysis was evaluated starting from the metaphysis on a length of 4 mm for each tibia; the top selection was chosen as the exact separation of the fibular head from the tibia. The cortical bone was outlined on transversal images, and the evaluation was performed on the whole bone area. The parameter used to assess the difference in density was the percent bone volume (PBV) BV/TV %. The percent bone volume represents the ratio of the segmented newly formed bone volume to the total value in the region of interest.

#### 2.6.2. Peri-Implant Bone Assessment

**Figure 4:**
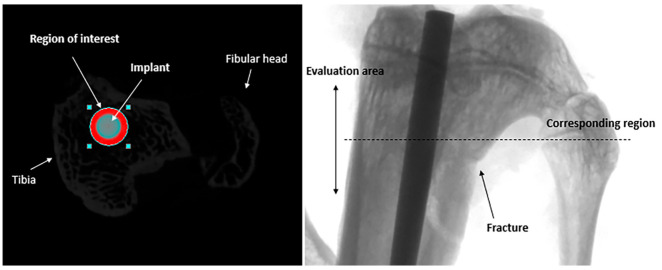
A transversal image (CTAnalyzer 1.13.0.0, Bruker, Belgium) of a tibial implant and the region of interest (ROI) with the corresponding frontal micro-Ct image.

The newly formed bone around the implant was evaluated through micro-CT scans. The results were obtained by defining, in bone, a circular region of interest with a 0.5 mm width around the implant (1 mm), as shown in Figure 4. The region of interest (ROI) was centered on the implant with a round shape in the metaphysis of the tibiae, and data were collected from the evaluation of a length of bone of 3 mm. The top selection image was chosen at the exact separation of the fibular head from the tibia, including the fracture site. The same investigator performed the evaluation in the blind group. The groups were compared using the percent bone volume (PBV) BV/TV (%) parameter. In the groups where a rat died and was removed from the research, another tibia was chosen at random for micro-CT analysis.

### 2.7. Mechanical Tests—“Pulling Out Test”

**Figure 5:**
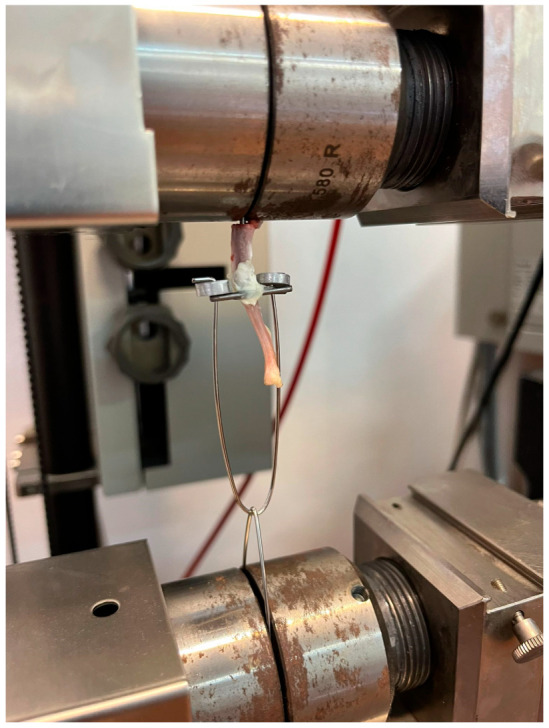
The pulling-out test on the Zwick Roell Z005 machine.

All the tibiae were sent to the Department of Materials Science and Engineering of the Technical University of Cluj-Napoca for implant removal and pull-force test measurement. Implant osseointegration was assessed through the pulling-out test, carried out on a Zwick Roell Z005 machine, with an accuracy class of 0.5. The tibiae were cut with care from the proximal epiphysis, leaving a length of 2 mm for gripping vertically in the pneumatic grips of the testing machine. Two identical steel rods of 1.5 mm in diameter and 20 mm in length were soldered perpendicular to the implant on the tibia, on a lower horizontal plane, using a Buildfix Pro UV curing composite (Schulzer), as shown in Figure 5. The two rods were used to pull downwards using a system that assures the 3D adjustment of the loading axis to the implant one. The employed rate for applying the load was 1 mm/min.

### 2.8. Histological Examination

After implant removal, tibial samples were prepared for histological evaluation. Tissues were fixed in 4% formaldehyde for 48 h, followed by decalcification in Biodec (Bio-Optica, Milano, Italy) over 30 days. The samples were then sectioned along the longitudinal axis using a microtome blade, ensuring the inclusion of the residual implant canal in each specimen. Samples were processed for dehydration in an automated tissue processor (Leica TP 1020) and subsequently embedded in paraffin wax. After cooling, specimens were sectioned to a thickness of 3 µm with a histological microtome. Hematoxylin Eosin and Trichrome Masson stains were applied for structural visualization. Examination and image capture were performed using a Leica DM 750 microscope equipped with a Leica ICC 50 HD video camera. For larger samples, multiple images were acquired and merged into a single composite image covering the entire implant canal using the Photo Merge function in Photoshop PS 6.

### 2.9. Statistical Analysis

The statistical analysis was carried out using GraphPad Prism Statistical Software version 9.3. Data were presented as values. The normal distribution of the values was determined using the Shapiro–Wilk test. One-way ANOVA was performed for normally distributed data to compare means among treatment groups at each time point. Two-way ANOVA was used to assess the effects of two independent factors—the treatment group (the state of the surface and existence or not of the LASER treatment) and the time of sacrifice—on the outcome of pull-force/micro-CT scan, where the rows were formed by the time of sacrifice. Tukey’s post hoc test was applied for significant ANOVA results (*p* < 0.05). The comparison of bone density in the ovariectomized vs. non-ovariectomized rats was performed using the Unpaired T-test. Statistical significance was set at *p* < 0.05.

## 3. Results

### 3.1. Micro-CT Scan

#### 3.1.1. Bone Density Assessment

**Figure 6:**
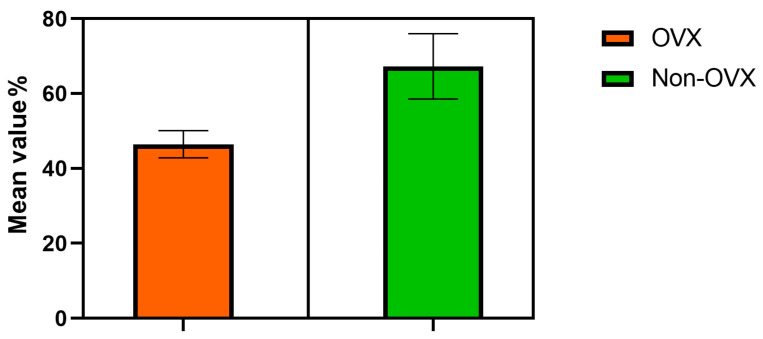
The bone density difference between ovariectomized (OVX) and non-ovariectomized (Non-OVX) rats: percent bone volume PV/TV % and mean and standard deviation.

Bone density was assessed by comparing eight tibias of four rats (two specimens were ovariectomized). The distribution was considered normal (the Shapiro–Wilk test). There was a significant difference (*p* = 0.04) in bone density between the ovariectomized (mean 46.45, std. deviation 3.616) and non-ovariectomized (mean 67.29, std. deviation 8.734) rats 12 weeks after surgery. Lower bone values were observed in the ovariectomized sample, as seen in Figure 6.

#### 3.1.2. Micro-CT Evaluation of Bone Density Peri-Implant

**Figure 7:**
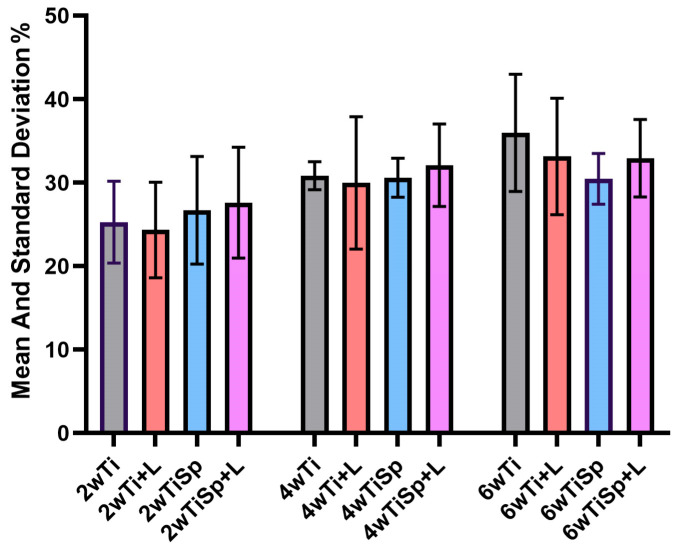
The mean and standard deviation graphics of all groups for the micro-CT scan (BV/TV %).

The Shapiro–Wilk test showed that the data are normally distributed in all groups. The one-way ANOVA tests performed for each time of sacrifice (2w, 4w, 6w) show no significant differences between treatment groups. The two-way ANOVA suggests that while time affects the outcome, the treatments themselves do not significantly affect the measured outcome, and no specific treatment stands out as significantly different across any time point. The means and standard deviations of all groups can be viewed in Table 1 and Figure 7.

### 3.2. Pull-Force Test Evaluation

**Figure 8:**
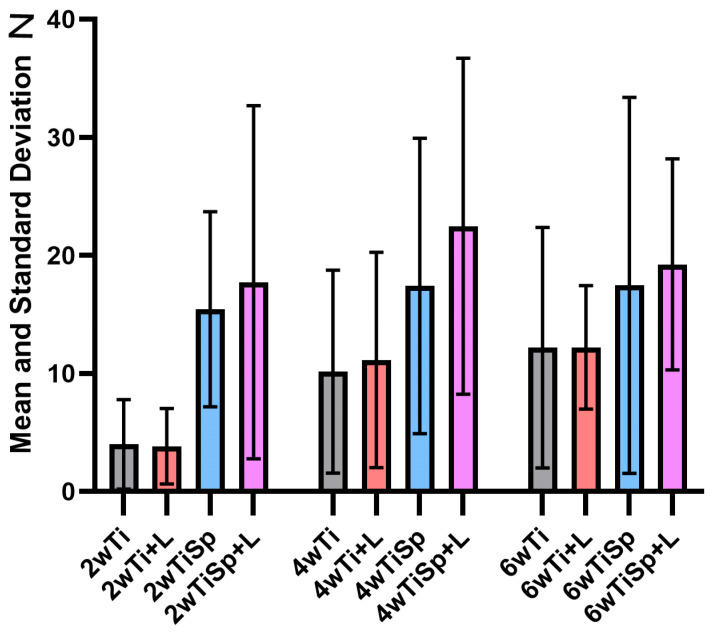
The mean and standard deviation graphics of all groups for the pull-force test (N).

The dataset was normally distributed in all groups (the Shapiro–Wilk test). The one-way ANOVA showed statistically significant differences in the 2-week group where the pull force of TiSp+L was superior to those of Ti (*p* = 0.0499) and Ti+L (*p* = 0.0463). No other statistically significant differences were observed at the 4- and 6-week sacrifice times. The means and standard deviations can be observed in Table 2 and Figure 8.

The two-way ANOVA revealed a significant effect of the treatment groups (Column Factor) on pull force (*p* = 0.0007), while neither the time points (Row Factor, *p* = 0.0977) nor the interaction between treatment and time (*p* = 0.9430) were statistically significant. TiSp+L had a significantly higher pull force compared to both Ti (*p* = 0.0047) and Ti+L (*p* = 0.0047). No other significant differences were observed, but the groups Ti and TiSp, (*p* = 0.0563) and Ti+L and TiSp (*p* = 0.0598) were close to statistical significance in favor of TiSp.

### 3.3. Histological Examination

**Figure 9:**
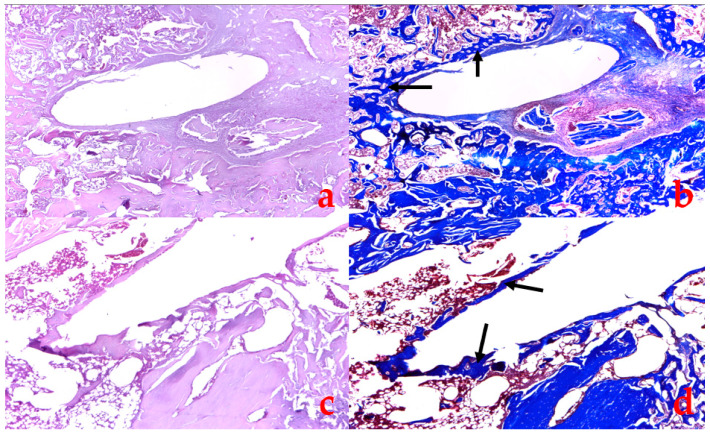
A histology slide using a Leica DM 750 microscope for the 4-week Ti+L (**a**,**b**) and TiSp+L (**c**,**d**) implants in Hematoxylin Eosin stain (**a**,**c**) and Trichrome Masson (**b**,**d**); the black arrows point towards newly formed bone tissue surrounding the implant.

In all groups sacrificed at the 2-week mark (2w), a conjunctive periosteal discontinued lamella formed around the implant, and the trabecular bone lamellae appeared thinned with an aspect of osteoporosis. The compact bone was not affected by low bone density in connection with the development of osteoporosis. There was no qualitative difference in bone formation between the 2w groups. After 2 weeks (4w groups), the peri-implant bone was superior in all groups compared to the 2w samples, and all of the implants were encased in a bony structure. The peri-implant bone lamellae were thicker in the LASER-irradiated groups. There was no difference between the types of implants with regard to bone formation, but LASER irradiation increased the bone quality, as shown in Figure 9. In the 6w groups, the quality of the peri-implant bone was better than in the 2w groups but similar to 4w (stagnation). The 6w LASER irradiated groups showed thicker bone tissue compared to the non-irradiated groups. The periosteal lamella did not change in any of the groups at 4 and 6 weeks compared to 2 weeks.

## 4. Discussion

To the best of our knowledge, this was the first study that evaluated the osseointegration of titanium implants with selectively anodized surface patterning and LASER therapy in a fractured osteoporotic bone. The effect of LASER therapy on bone formation has been observed through many studies; however, the used parameters differed. At present, there is no consensus on the parameters used for LASER photobiomodulation. Investigators achieved significant results using different wavelengths (940 to 810 nm), times of exposure (80 to 402 s), fluencies (5 to 86 J/cm^2^), and numbers of sessions (7, with one every other day, or 10 consecutive days) [18,19,20,21]. Among these, fluency (J/cm^2^) is the most employed parameter for establishing the protocol, as it shows the energy transmitted to the target surface. The studies conducted by Gomes and Masotti show that osseointegration was superior in rodents if the fluency was above 20 J/cm^2^ [20,22]. We used the protocol that we implemented in the pilot study, with a higher fluency (80 J/cm^2^), similar to Campanha et al. [19,20,23] for an enhanced bio-stimulatory effect. On the other hand, the strategy behind the applied novel surface patterning of implants was to offer both zones for bone microtubules to grow into (the cellular selectively anodized areas) and zones that assure a macroscopic anchoring of press-fit type in the medullar canal (the smooth zones), leading to improved implant fixation in the short and long term. The present study was conceived to analyze the synergistic effect of photobiomodulation and this type of surface patterning in the difficult case of intramedullary implants used for assisting fracture healing of osteoporotic bone.

Osteoporosis affects the spongious (trabecular) bone more than the cortical bone, increasing the probability of bone fracture. In our results, a clear bone density difference could be observed after comparing healthy rats to the ovariectomized ones, 12 weeks after surgery. (*p* = 0.04). This was the reason for studying the synergistic effect of LASER therapy and implant texture on ovariectomized rats, with a very unfavorable case in terms of bone strength and healing process. As a functional assessment of the tested implantation/post-implantation procedure, the pull-force test enhanced the osseointegration of the surface-patterned implants for all sacrifice time groups. This was a confirmation of the patterning strategy using an axial alternation of smooth and cellular morphology for the surfaces of implants. The cellular rough sheets grown as cylindrical segments along the axis of the implant were proven to act like anchors for newly formed bone protrusions, while the smooth ones had the effect of macroscopic blocking inside the intramedullary canal. However, a statistically significant difference was observed only at 2 weeks when surface patterning was associated with LASER therapy. For the groups that were sacrificed at 4 and 6 weeks after finalizing the procedure, neither surface patterning, LASER therapy, nor their cumulative effect showed any statistically significant difference, although their mean difference indicated a positive effect. The two-way ANOVA that we used to compare all the groups showed that time exerted no statistical effect on the bone/implant interaction, but it showed that the groups with surface patterning combined with LASER therapy were superior to the groups with unmodified titanium implants, with a statistical significance. Furthermore, the cumulative pull-force test for the groups that received an implant with surface patterning yielded better results compared to the groups with unmodified titanium (TiSp vs. Ti *p*= 0.0563, TiSp vs. Ti+L *p* = 0.0598). Previous research suggests that the bio-stimulatory effects of LASER therapy are most effective during the early stages of bone healing, including the inflammatory, angio-mesenchymal, and soft callus formation phases [5]. This could explain why we observed significant results in the group treated within the first two weeks, as the early intervention maximized the regenerative benefits of LASER therapy during these critical stages of bone repair. Similar results were also observed in a study conducted by de Vasconcellos et al. that evaluated osseointegration in a similar population of rats at the same time of sacrifice [24].

The micro-CT morphological evaluation of all groups led to the conclusion that bone quality was better in the groups that received LASER therapy sacrificed at 4 weeks, with no relevant changes for the 6-week groups, and the implant type made no difference.

In a previous pilot study conducted by our group, using non-ovariectomized subjects following identical surgical procedures on unmodified titanium implants and the same LASER protocol, a significant increase in pull-force (N) was observed in the groups treated with LLLT at the times of sacrifice [23]. We consider that osteoporosis exerted an important detrimental influence on bone formation and osseointegration.

Our study proposed a novel surface patterning of endosseous implants, with alternating cellular-shaped zones and smooth ones. The inner dimension of cells was around 50 nm, similar to the one found in the literature to promote early osteogenic differentiation and osteoblastic activity [17]. Although the employed technology could lead to the growth of titania nanotubes perpendicular to the surface of implants, with many advantages in terms of creating the possibility to insert antibacterial drugs, for instance, we have chosen to maintain the early cellular structuring on top of titanium implants, considering the effectiveness during the press-fit insertion. The overall macro-organization of the surface contributes to a highly durable structure for endosseous implants [25].

When considering the results of histological examination, we can state that LASER therapy offered the possibility for osseointegration to be more advanced, with a better quality of bone, but this was not effective enough in terms of interfacial strength due to the insufficient dynamic of hard tissue protrusions.

The subjects that we tested were osteoporotic after ovariectomy and fracture; thus, the therapy that targeted bone healing and osteosynthesis was performed for a difficult-to-treat group, mimicking the challenging clinical cases that may occur in human patients.

Our findings indicate that LASER therapy enhances local metabolism and promotes cell proliferation, effectively supporting the early stages of bone healing. Additionally, the proposed surface patterning improves bone anchoring through a mechanism involving both nanotubule ingrowth and macro-fixation, suggesting that these approaches work synergistically to optimize bone integration and stability.

## 5. Conclusions

As observed in rats, low-level LASER therapy (LLLT) combined with surface patterning of titanium implants through selective anodization and heat treatment enhanced the osseointegration process in osteoporotic bones, with the most pronounced effects being observed during the initial phases of bone healing. The synergistic approach that we proposed is expected to optimize implant osseointegration for human patients by accelerating cellular responses and promoting early bone regeneration.

## Figures and Tables

**Table 1:** The means and standard deviations of each group after the Micro-CT evaluation.

Time of Sacrifice (Weeks)	Ti (Mean ± SD) %	Ti+L (Mean ± SD) %	TiSp (Mean ± SD) %	TiSp+L (Mean ± SD) %
2 Weeks	25.26 ± 4.90	24.33 ± 5.73	26.70 ± 6.45	27.60 ± 6.64
4 Weeks	30.83 ± 1.67	29.97 ± 7.93	30.60 ± 2.33	32.10 ± 4.94
6 Weeks	35.97 ± 7.01	33.14 ± 6.98	30.46 ± 3.02	32.92 ± 4.64

**Table 2:** The means and standard deviations of each group during the pull-force evaluation (N).

Time of Sacrifice (Weeks)	Ti (Mean ± SD) (N)	Ti+L (Mean ± SD) (N)	TiSp (Mean ± SD) (N)	TiSp+L (Mean ± SD) (N)
2 Weeks	3.99 ± 3.77	3.83 ± 3.18	15.44 ± 8.26	17.73 ± 14.96
4 Weeks	10.15 ± 8.58	11.14 ± 9.12	17.41 ± 12.50	22.46 ± 14.23
6 Weeks	12.18 ± 10.18	12.21 ± 5.22	17.46 ± 15.93	19.23 ± 8.94

## Data Availability

The original contributions presented in the study are included in the article, further inquiries can be directed to the corresponding author.
